# Co-frequency or contrary? The effects of Qiwei Baizhu Powder and its bioactive compounds on mucosa-associated microbiota of mice with antibiotic-associated diarrhea

**DOI:** 10.3389/fcimb.2024.1483048

**Published:** 2024-10-28

**Authors:** Zeli Zhang, Yan Yang, Yingsi Zhang, Guozhen Xie

**Affiliations:** School of Pharmacy, Hunan University of Chinese Medicine, Changsha, China

**Keywords:** Qiwei Baizhu Powder, total glycosides, diarrhea, mucosa-associated microbiota, immunity

## Abstract

Qiwei Baizhu Powder (QWBZP) has been proven effective in treating antibiotic-associated diarrhea (AAD), and the mechanism is associated with regulating the gut microbiota. However, the role of the bioactive compounds of QWBZP in regulating the gut microbiota is still unclear. In this study, 24 mice were divided into a normal control group (N), a model group (R), a QWBZP decoction group (TW), and a QWBZP-TG group (TG). AAD mouse models were established by mixed antibiotic administration. After modeling, mice in the TW group and TG group were treated with QWBZP decoction and QWBZP-TG, respectively. Mice in the N group and R group were gavaged with sterile water. 16S rRNA gene sequencing was used to investigate the changes of mucosa-associated microbiota (MAM) in the small intestine of mice. Moreover, the levels of diamine oxidase (DAO), D-Lactate, secretory immunoglobulin A (sIgA), interleukin 6 (IL-6), IL-10, and tumor necrosis factor-α (TNF-α) were detected using enzyme-linked immunosorbent assay (ELISA) kits. The results showed that QWBZP-TG significantly altered the diversity, structure, and abundance of MAM in the AAD mice. QWBZP-TG exerted a stronger suppression effect on *Escherichia* and *Clostridium* compared with QWBZP decoction. Meanwhile, QWBZP-TG downregulated the abundance of *Lactobacillus*, which elicited an opposite effect to QWBZP decoction. *Prevotella* was the signature bacteria that responded to the QWBZP-TG intervention. Furthermore, both QWBZP decoction and QWBZP-TG decreased the levels of DAO, D-Lactate, sIgA, IL-6, and TNF-α in the AAD mice. The role of glycosides is to help QWBZP ameliorate diarrhea symptoms by inhibiting the proliferation of diarrhea-associated bacteria, reducing inflammation and regulating immunity.

## Introduction

Traditional Chinese medicine (TCM) formula, usually composed of two or more Chinese materia medica, is a primary approach to cure diseases in Chinese medicine. The mechanism of the TCM formula in the treatment of diseases is involved in multi-components, multi-targets, and multi-paths. Decoction is the main form to apply TCM in practice and is rich in small-molecule compounds, glycosides, and polysaccharides. Small-molecule compounds are considered pharmacodynamic ingredients for their high bioavailability. However, glycosides and polysaccharides are commonly ignored due to their poor absorption (Shen et al., 2018). Many studies have revealed that glycosides and polysaccharides are difficult to digest in the small intestine, but they still exhibit immunomodulatory (Zeng et al., 2024), anti-inflammatory (Sun et al., 2024), anti-diabetic (Wang et al., 2023), and anti-cancer effects (Ji et al., 2020). Thus, it is important to explore the contributions of different bioactive components to the therapeutic effects of TCM formula.

Qiwei Baizhu Powder (QWBZP) is a classical formula for infantile diarrhea treatment. QWBZP is now widely used to treat gastrointestinal diseases, including antibiotic-associated diarrhea (AAD), functional diarrhea, and irritable bowel syndrome. Previous studies have revealed that QWBZP could improve AAD symptoms by regulating the gut microbiota and exerting anti-inflammatory effects (Hui et al., 2020; Long et al., 2020). Ultra-high performance liquid chromatography quadrupole time-of-flight tandem mass spectrometry (UHPLC-Q-TOF-MS/MS) results showed that glycosides were the dominant components of QWBZP decoction (Xie et al., 2022b). Recently, we demonstrated that the total glycoside (TG) from QWBZP decoction (QWBZP-TG) also ameliorated diarrhea symptoms induced by antibiotics (Xie et al., 2021). However, the anti-diarrheal mechanism of QWBZP-TG has not been addressed.

Gut microbiota is a complex ecosystem consisting of bacteria, viruses, and fungi. Exogenous substances, such as diet and medication, disturb the intestinal microecology and change the microbial composition. Gut microbiota secretes numerous enzymes and efficiently metabolizes food and drugs (Tian et al., 2022). In addition, the gut possesses 80% of immune cells and is an important immune organ. Microorganisms and their metabolites play an essential role in maintaining immune regulation. Disruption of immune signaling pathways causes chronic inflammation and tissue damage, which have become common features of non-infectious diarrhea (Hosseinkhani et al., 2021). Previous research has shown that ginsenosides relieved the immune disorder in cyclophosphamide-induced mice by regulating the gut microbiota (Zhou et al., 2021). Astragaloside IV ameliorated experimental autoimmune myasthenia gravis by regulating CD4+ T cells and altering the gut microbiota (Weng et al., 2023). Therefore, we hypothesized that the anti-diarrheal mechanism of QWBZP-TG is related to regulating the gut microbiota and immunity.

The gut has two distinct microbial ecosystems, namely, lumen-associated microbiota and mucosa-associated microbiota (MAM). MAM refers to the microorganisms that adhere to the mucous layer of the intestinal tract with relatively stable structure and composition (Shi et al., 2017). MAM is regarded as an intestinal barrier that assists in colonization resistance, resisting the invasion of pathogenic bacteria, and regulating the host immunity under normal conditions (Shi et al., 2017). Previous studies revealed that MAM was more sensitive and characteristic in response to exogenous stressors and gastrointestinal physiological functions (Zhang et al., 2020; Juge, 2022). Impaired barrier function is associated with intestinal diseases. Dysbacteriosis destroys the barrier function, increases intestinal permeability, transfers intestinal contents from the cavity to the mucous layer, activates the host immunity, and then induces immune diseases or inflammatory diseases (Wang et al., 2021). In this study, we investigated the effects of QWBZP-TG on the gut microbiota, inflammation, and immunity in the AAD mice. The objectives of this study were to (1) clarify how QWBZP-TG might affect the MAM and immunity of AAD mice and (2) explain the contribution of TGs to QWBZP decoction in AAD treatment.

## Materials and methods

### Materials and reagents

QWBZP consists of seven Chinese herbal slices, as shown in **Table 1**. All the Chinese herbal slices were purchased from the First Hospital of Hunan University of Chinese Medicine and certified by Prof. Qingping Pan from the School of Pharmacy of Hunan University of Chinese Medicine. A mixture of antibiotics consists of gentamicin sulfate (01Y07011A2, Yichang Renfu Pharmaceutical Co. Ltd., Yichang, China) and cefradine (06200502, Jilin Wantong Pharmacy Group Co., Jilin, China). Enzyme-linked immunosorbent assay (ELISA) kits of diamine oxidase (DAO, JM-02511M1), D-Lactate (JM-11364M1), interleukin 6 (IL-6, JM-02446M1), IL-10 (JM-02459M1), tumor necrosis factor-α (TNF-α, JM-02415M1), and secretory immunoglobulin A (sIgA, JM-02713M1) were purchased from Jingmei Biotechnology Co. Ltd. (Jiangsu, China).

**Table 1:** The components of QWBZP.

Herbal name	Latin name	Place of origin (China)	Batch number	Amount used (g)
Bai-zhu	Atractylodis Macrocephalae Rhizoma	Hunan	20201014	15
Ren-shen	Ginseng Radix et Rhizoma	Jilin	20200610	7.5
Fu-ling	Poria	Hunan	20201109	15
Ge-gen	Puerariae Lobatae Radix	Hunan	20201020	15
Mu-xiang	Aucklandiae Radix	Yunnan	20201011	6
Huo-xiang	Pogostemonis Herba	Guangdong	20200816	15
Gan-cao	Glycyrrhizae Radix et Rhizoma	Neimenggu	20200610	3

### Preparation of QWBZP and QWBZP-TG

QWBZP herbal slices were boiled twice with distilled water (1:10, *w*/*v*) for 30 min each time. The filtrate was combined and concentrated until the final crude drug concentration was 0.34 g/ml. QWBZP-TG was prepared in accordance with our optimized method (Xie et al., 2021). The composition of QWBZP-TG is shown in **Supplementary Table S1**. QWBZP-TG powder was dissolved in sterile water and mixed homogeneously. The concentration of QWBZP-TG solution was 6.31 mg/ml. The QWBZP decoction and QWBZP-TG solution were stored at 4°C and were reheated at 30°C before use.

### Animals and feed

All mouse works followed the recommendations of the National Research Council Guide for the Care and Use of Laboratory Animals, with the protocols approved by the Animal Care and Use Committee of Hunan University of Chinese Medicine (authorization number: LL2020102103). Male Kunming mice (specific pathogen-free, SPF), 5-week-old, weighing 18–22 g, were provided by Hunan Slaccas Jingda Laboratory Animal Co., Ltd. (Changsha, China) with license number SCXK (Xiang) 2019-0004. Mice were maintained in an SPF animal facility (12-h light/dark cycle, 22°C–25°C, 45%–55% relative humidity) with free access to standard laboratory diet and water during the experimental period.

### Experimental design

**Figure 1:**
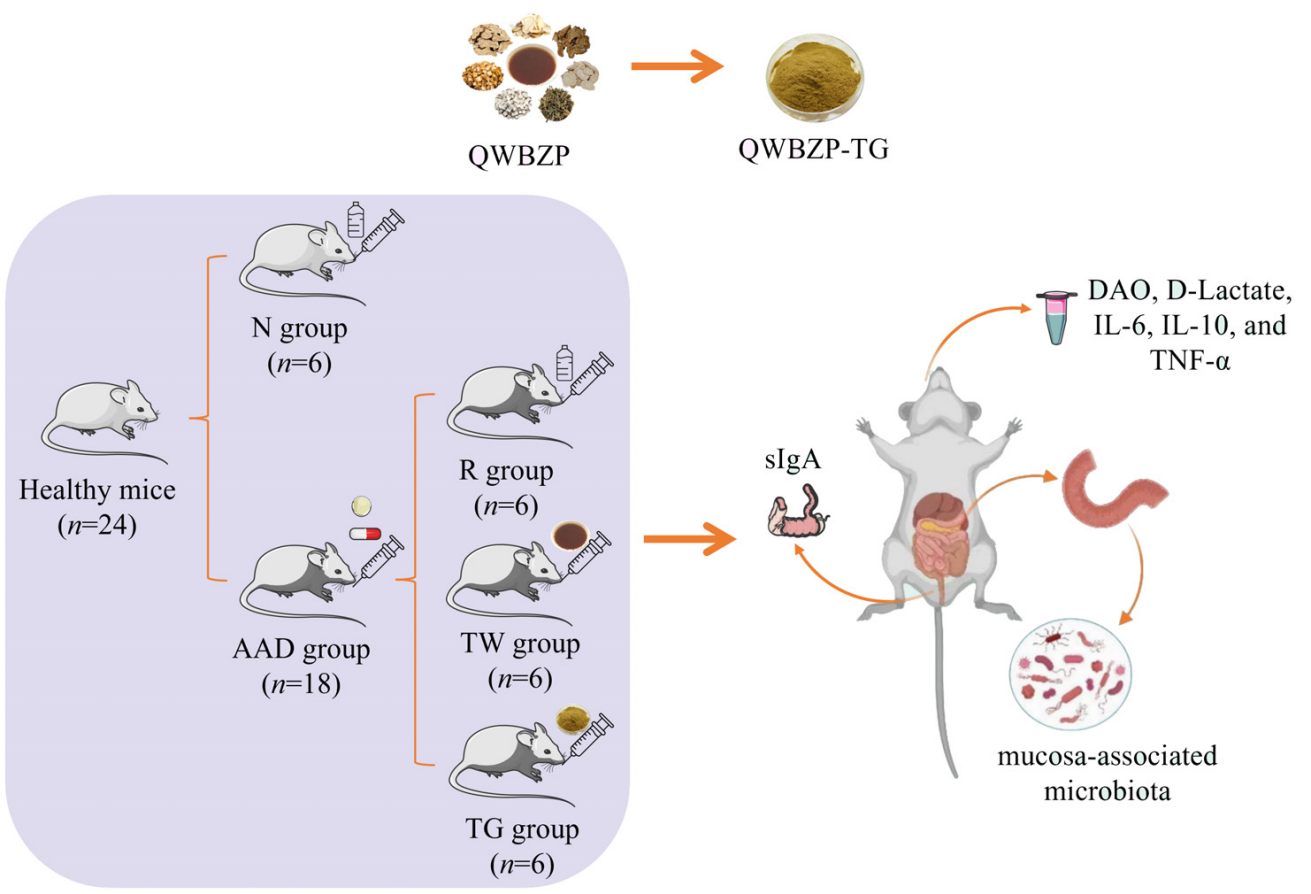
Experimental flow chart. QWBZP, Qiwei Baizhu Powder; QWBZP-TG, total glycoside of QWBZP; N, normal control; AAD, antibiotic-associated diarrhea; R, model; TW, QWBZP decoction; TG, QWBZP-TG; DAO, diamine oxidase; IL-6, interleukin 6; IL-10, interleukin 10; TNF-α, tumor necrosis factor-α; sIgA, secretory immunoglobulin A.

After adaptive feeding, 24 mice were randomly divided into a normal control group (N, *n* = 6) and an AAD group (*n* = 18). The AAD mice were administered a mixture of gentamycin sulfate and cefradine (62.5 mg/ml, 0.4 ml) twice per day for five days to establish AAD models (Hui et al., 2020). Normal control mice were administered sterile water. After 5-day modeling, the AAD mice were further randomly divided into three groups (*n* = 6): (1) model group (R), treated with sterile water; (2) QWBZP decoction group (TW), treated with QWBZP at 9.945 g/kg (body weight) [BW]/d; (3) QWBZP-TG group (TG), treated with QWBZP-TG at 147.2 mg/kg (BW)/d. The dosage of QWBZP decoction was calculated from the equivalent conversion of the body weight between mice and humans. The dosage of QWBZP-TG was equal to the clinical dose of QWBZP multiplied by the extraction rate of QWBZP-TG (Xie et al., 2021). The administered volume was 0.4 ml for each mouse, twice per day for three days. The experimental design is exhibited in **Figure 1**.

### Sample preparation

After treatment, blood was collected by the eyeball extraction method and left at room temperature standing for 2 h, then centrifuged at 4°C (3500 rpm, 15 min) to isolate serum. The obtained serum samples were stored at −80°C until analysis for DAO, D-Lactate, IL-6, IL-10, and TNF-α using ELISA kits. Subsequently, the mice were sacrificed by cervical dislocation. Under aseptic conditions, the small intestine (from duodenum to ileum) was collected and split longitudinally. Residue in the intestinal cavity was rinsed with normal saline, and then intestinal mucosa was collected by sterile coverslips (Li et al., 2022). All mucosal samples were immediately placed in sterile cryopreservation tubes, frozen in liquid nitrogen, and stored at −80°C until DNA extraction. Colon mucosal samples were collected using the same approach. The colon mucosal samples and normal saline (1:9, *w*/*v*) were vortex-mixed and centrifuged at 4°C (3500 rpm, 10 min) to obtain supernatant. Then supernatants were stored at −80°C until analysis for sIgA using ELISA kits.

### Biochemical analysis

The levels of DAO, D-Lactate, IL-6, IL-10, TNF-α, and sIgA were determined using corresponding ELISA kits, respectively. Standard blanks and sample wells were prepared according to manufacturer instructions.

### 16S rRNA gene sequencing and bioinformatic analysis

Genomic DNA was extracted from the small intestinal mucosa using a DNA isolation kit (Omega, Norcross, GA, USA) and evaluated using a Nano-drop 2000 (Thermo Fisher Scientific, Waltham, Massachusetts, USA). The V3–V4 regions of the 16S rRNA genes of bacteria in mucosal samples were amplified by PCR using the following primers: 338F (5’-ACTCCTACGGGAGGCAGCAG-3’) and 806R (5’-GGACTACHVGGGTWTCTAAT-3’). Amplicon pyrosequencing was performed on the DNA samples using the Illumina MiSeq platform (Illumina, San Diego, CA, USA).

Demultiplexed sequences from each sample were quality filtered and trimmed, de-noised, and merged, and then the chimeric sequences were identified and removed using the Quantitative Insights into Microbial Ecology version 2 (QIIME2) dada2 to obtain clean sequences. High-qualified tags with ≥97% similarity were clustered into the same operational taxonomic units and classified against the Greengenes v138 database. Alpha diversity indices (Chao1, Faith’s phylogenetic diversity (Faith_pd), Shannon, and Simpson) were calculated using QIIME2. Beta diversity analysis was performed using nonmetric multidimensional scaling (NMDS) based on the Bray-Curtis distance. Linear discriminant analysis effect size (LEfSe, LDA score = 4) was conducted to identify microbial structure differences among groups based on genus level.

### Data analysis

Data were statistically analyzed using SPSS 26.0 and expressed as the mean ± standard deviation (SD). Statistical analysis was performed using the Mann–Whitney–Wilcoxon and Kruskal–Wallis tests. *P* < 0.05 was considered statistically significant. Bar charts were generated using Origin 2022.

## Result

### Effect of QWBZP-TG on the diversity of MAM in the AAD mice

**Figure 2:**
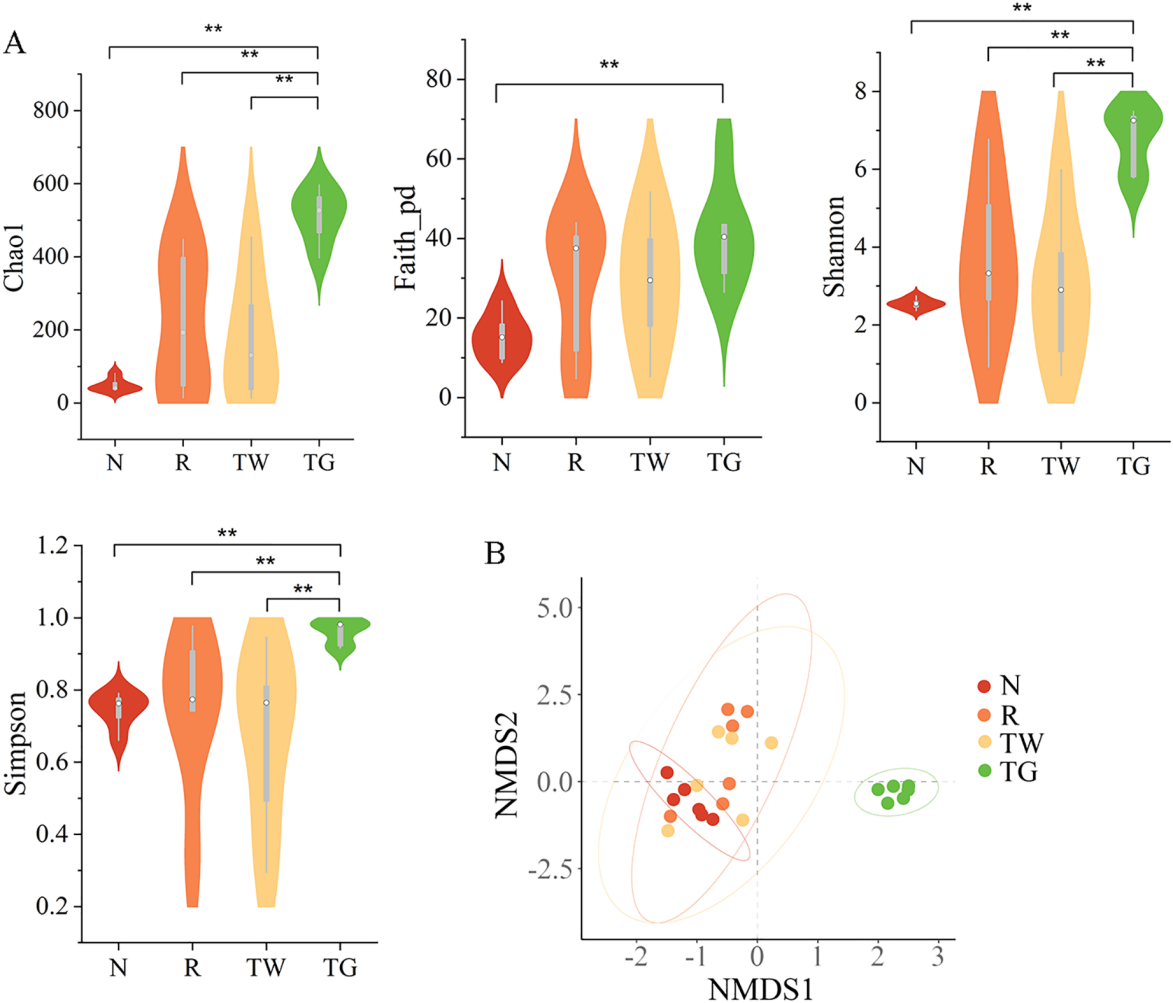
Effects of QWBZP-TG on the microbial diversity in the AAD mice. **(A)** Alpha diversity (Chao1, Faith_pd, Shannon, and Simpson); **(B)** Beta diversity. Nonmetric multidimensional scaling (NMDS) analysis based on Bray-Curtis distance. N, normal control group; R, model group; TW, QWBZP decoction group; TG, QWBZP-TG group. Data are expressed as mean ± standard deviation (SD), *n* = 6. ^**^ *P* < 0.01.

The alteration of MAM diversity in the small intestine was assessed. The result showed that the alpha diversity indices (Chao1, Faith_pd, Shannon, and Simpson) of the R group, TW group, and TG group were higher than those of the N group (**Figure 2A**). The differences in the alpha diversity index among the four groups were analyzed using Wilcoxon. Compared with the N group, QWBZP-TG intervention significantly increased the richness (*P*_Chao1_ = 0.002 and *P*_Faith_pd_ = 0.002) and evenness (*P*_Shannon_ = 0.002 and *P*_Simpson_ = 0.002) of MAM in the small intestine. Meanwhile, QWBZP-TG treatment significantly increased Chao1 (*P* = 0.009) of MAM in the AAD mice. The results prompted that antibiotics, QWBZP decoction, and QWBZP-TG intervention disturbed the intestinal microecology in mice.

### Effect of QWBZP-TG on the structure of MAM in the AAD mice

NMDS based on Bray_Curtis distance was carried out to determine the differences in bacterial community structure among the four groups. The bacterial community structure of the TG group remarkably diverged from that of the N, R, and TW groups (**Figure 2B**, Stress = 0.103, F = 5.684, *P* = 0.001). However, the bacterial community structure of the R and TW groups could not be distinguished significantly. These results indicated that QWBZP-TG notably altered the structure of MAM. Intriguingly, the effect of QWBZP-TG on bacterial structure was more potent than that of QWBZP decoction, as evident from the structure of MAM was different from that of the normal control group after QWBZP-TG administration.

### Effect of QWBZP-TG on the abundance of MAM in the AAD mice

**Figure 3:**
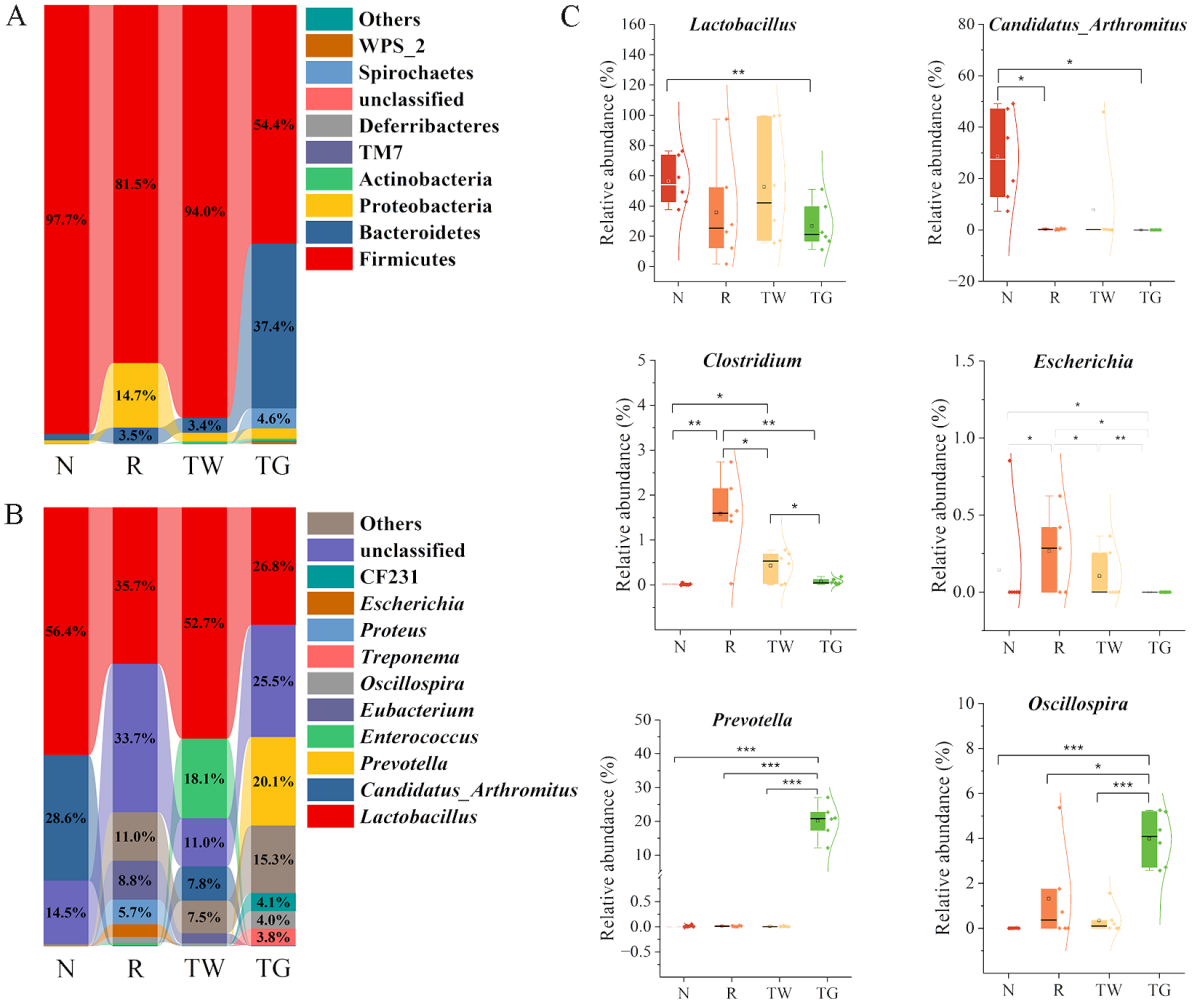
Effects of QWBZP-TG on the microbial composition in the AAD mice. **(A)** Relative abundance at the phylum level; **(B)** Relative abundance at the genus level. Data are expressed as the mean relative abundance. **(C)** Relative abundance of specific genera. N, normal control group; R, model group; TW, QWBZP decoction group; TG, QWBZP-TG group. Data are expressed as mean ± standard deviation (SD). ^*^ *P* < 0.05, ^**^ *P* < 0.01, and ^***^ *P* < 0.001.

Firmicutes, Bacteroidetes, and Proteobacteria were the dominant phyla in the MAM of mice. The R group showed a 16.2% decrease in the relative abundance of Firmicutes and a 13.8% increase in the relative abundance of Proteobacteria compared with the N group (**Figure 3A**). In comparison, the relative abundance of Firmicutes and Proteobacteria in the TW group was close to those in the N group. Nevertheless, the TG group exhibited an observable reduction in Firmicutes abundance while a marked increase in Bacteroidetes abundance compared with the N group and the R group. These findings suggested that QWBZP decoction and QWBZP-TG showed different effects on the bacteria phyla of MAM.

Genus-level analysis revealed a marked shift in the taxonomic distribution of the MAM after mixed antibiotics, QWBZP decoction, and QWBZP-TG intervention. These interventions dramatically altered the abundance of *Lactobacillus* and *Candidatus_Arthromitus*, two dominant genera that kept intestinal bacteria balanced in the normal mice (**Figure 3B**). The abundance of *Lactobacillus* and *Candidatus_Arthromitus* (*P* = 0.012) decreased after mixed antibiotic administration (**Figure 3C**). Treatment with QWBZP decoction upregulated the *Lactobacillus* abundance. Conversely, QWBZP-TG did not recover the abundance of the *Lactobacillus* and *Candidatus_Arthromitus* in the AAD mice. These results indicated that QWBZP decoction was more effective in recovering the symbiotic bacteria in the small intestinal mucosa of AAD mice. *Escherichia* and *Clostridium*, two diarrhea-associated genera, were enriched in the AAD mice, and both were suppressed by QWBZP decoction and QWBZP-TG (**Figure 3C**). In particular, *Escherichia* was not detected in the mice of the TG group, suggesting that QWBZP-TG exhibited a stronger suppressed effect on diarrhea-associated genera than QWBZP decoction. Meanwhile, the TG group showed a noticeable rise in the relative abundance of *Prevotella* and *Oscillospira* (**Figure 3C**).

**Figure 4:**
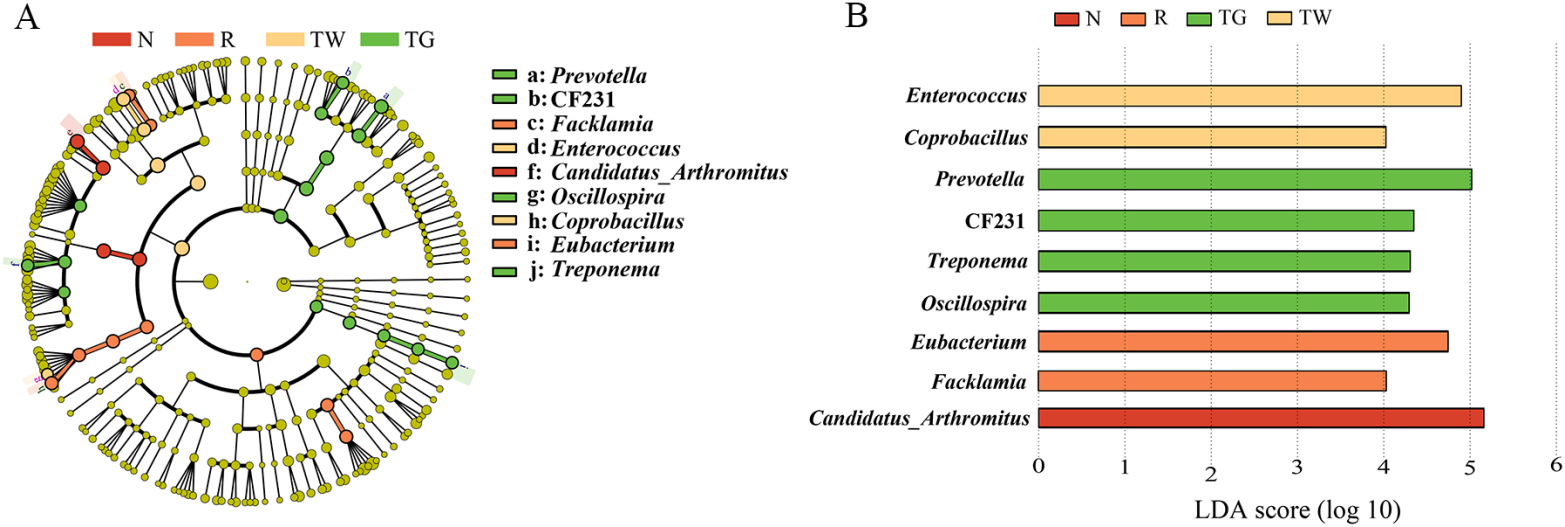
Characteristics bacteria of the four groups. **(A)** Cladogram of linear discriminant analysis effect size (LEfSe) analysis at the genus level. **(B)** Linear discriminant analysis (LDA) score (LDA > 4) of core differential bacteria (at the genus level). N, normal control group; R, model group; TW, QWBZP decoction group; TG, QWBZP-TG group.

According to LEfSe analysis (**Figures 4A, B**), *Eubacterium* (LDA = 4.74, *P* = 0.0066) and *Facklamia* (LDA = 4.03, *P* = 0.0013) were the characteristic genera in the AAD mice. *Enterococcus* (LDA = 4.90, *P* = 0.0058) and *Coprobacillus* (LDA = 4.02, *P* = 0.0093) were relatively enriched after QWBZP decoction treatment. QWBZP-TG intervention notably increased the abundance of *Prevotella* (LDA = 5.02, *P* = 0.0025), CF231 (LDA = 4.35, *P* = 0.0001), *Oscillospira* (LDA = 4.29, *P* = 0.0081), and *Treponema* (LDA = 4.31, *P* = 0.0000).

### Effect of QWBZP-TG on the function of MAM in the AAD mice

**Figure 5:**
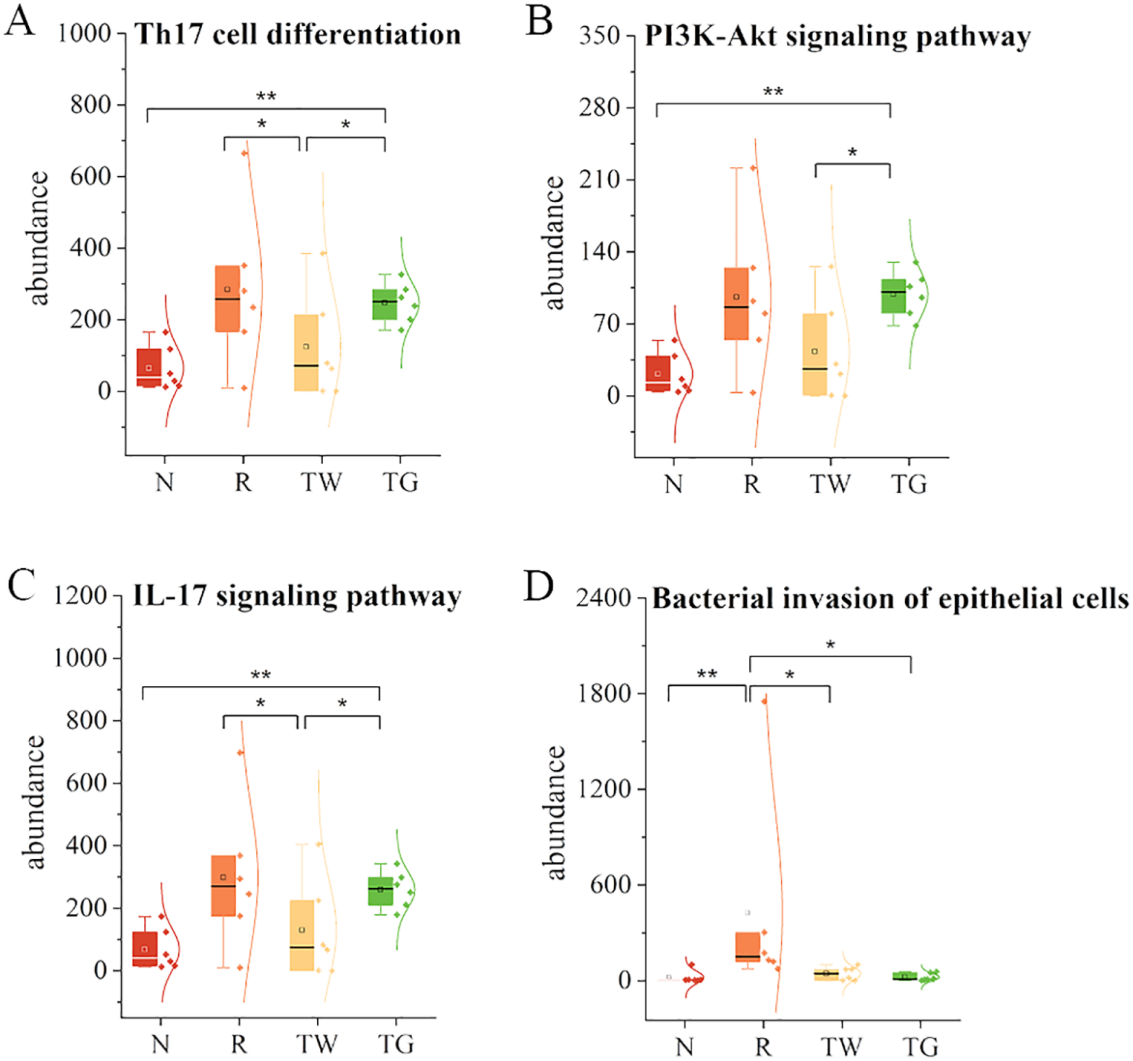
Functional prediction of mucosa-associated microbiota. **(A)** Th17 cell differentiation; **(B)** PI3K-Akt signaling pathway; **(C)** IL-17 signaling pathway; **(D)** Bacterial invasion of epithelial cells. N, normal control group; R, model group; TW, QWBZP decoction group; TG, QWBZP-TG group. Data are expressed as mean ± standard deviation (SD). ^*^ *P* < 0.05 and ^**^ *P* < 0.01.

According to network pharmacology analysis, the mechanisms of QWBZP in the treatment of diarrhea may be related to repairing damaged intestinal mucosa and regulating inflammatory signaling pathways, such as nuclear factor kappa-B (NF-κB), mitogen-activated protein kinase (MAPK), and the Janus kinase/signal transducer and activator of tran-ions (JAK/STAT) (Huang et al., 2022). To explore the association among QWBZP/QWBZP-TG, MAM, intestinal mucosal barrier, and inflammation, we first predicted the functional characteristic pathways of the Kyoto encyclopedia of genes and genomes (KEGG) from the 16S rRNA gene profile through phylogenetic investigation of communities by reconstruction of unobserved states (PICRUSt) analysis. In this study, we focused on the changes in infection, immune system, and signal transduction. The results showed that mixed antibiotics upregulated the pathways of T-helper cell 17 (Th17) cell differentiation (**Figure 5A**), phosphatidylinositol 3 kinase (PI3K)-protein kinase (Akt) signaling pathway (**Figure 5B**), IL-17 signaling pathway (**Figure 5C**), and bacterial invasion of epithelial cells (**Figure 5D**). Both QWBZP decoction and QWBZP-TG regulated these pathways. However, the regulation effects of QWBZP-TG on Th17 cell differentiation, PI3K-Akt signaling pathway, and IL-17 signaling pathway were weaker than that of QWBZP decoction.

### Effect of QWBZP-TG on the levels of DAO, D-Lactate, sIgA, IL-6, IL-10, and TNF-α in the AAD mice

**Figure 6:**
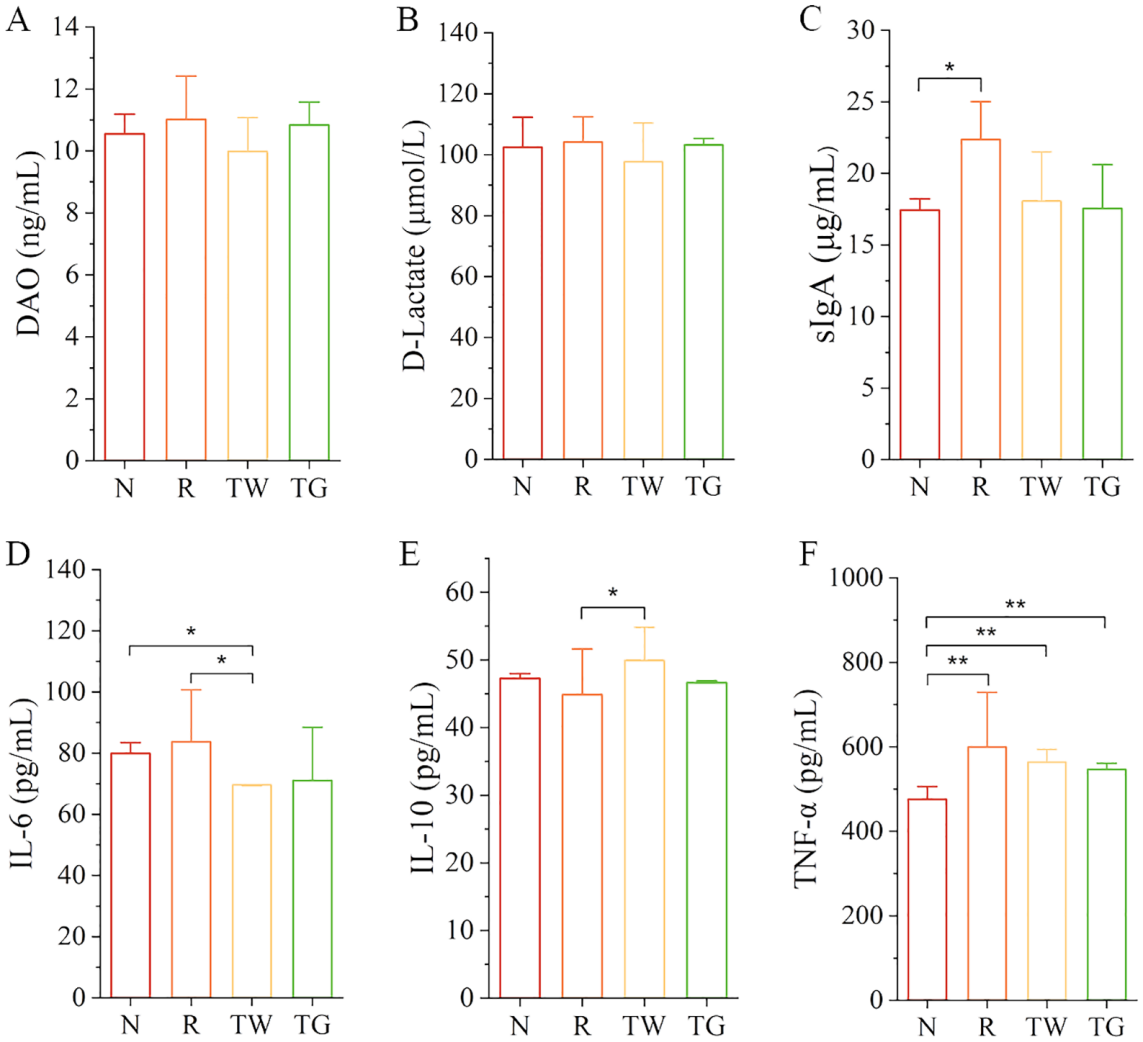
The levels of DAO, D-Lactate, sIgA, IL-6, IL-10, and TNF-α in the four groups. **(A)** DAO; **(B)** D-Lactate; **(C)** sIgA; **(D)** IL-6; **(E)** IL-10; **(F)** TNF-α. N, normal control group; R, model group; TW, QWBZP decoction group; TG, QWBZP-TG group. Data are expressed as mean ± standard deviation (SD). ^*^ *P* < 0.05 and ^**^ *P* < 0.01.

To investigate the impacts of QWBZP-TG on intestinal mucosal barrier and inflammation in the AAD mice, we detected mucosal barrier indices (DAO and D-Lactate), immune factor (sIgA), and cytokines (IL-6, IL-10, and TNF-α). The data revealed that mixed antibiotics upregulated the levels of DAO, D-Lactate, sIgA, IL-6, and TNF-α while downregulated the level of IL-10 (**Figure 6**). Importantly, QWBZP decoction and QWBZP-TG regulated these indices. Moreover, the regulation effects on IL-6 and IL-10 were more significant in the case of QWBZP decoction. These results were consistent with the predicted results mentioned above to some extent.

### Correlation analysis of the specific bacterial genera with DAO, D-Lactate, sIgA, IL-6, IL-10, and TNF-α

**Figure 7:**
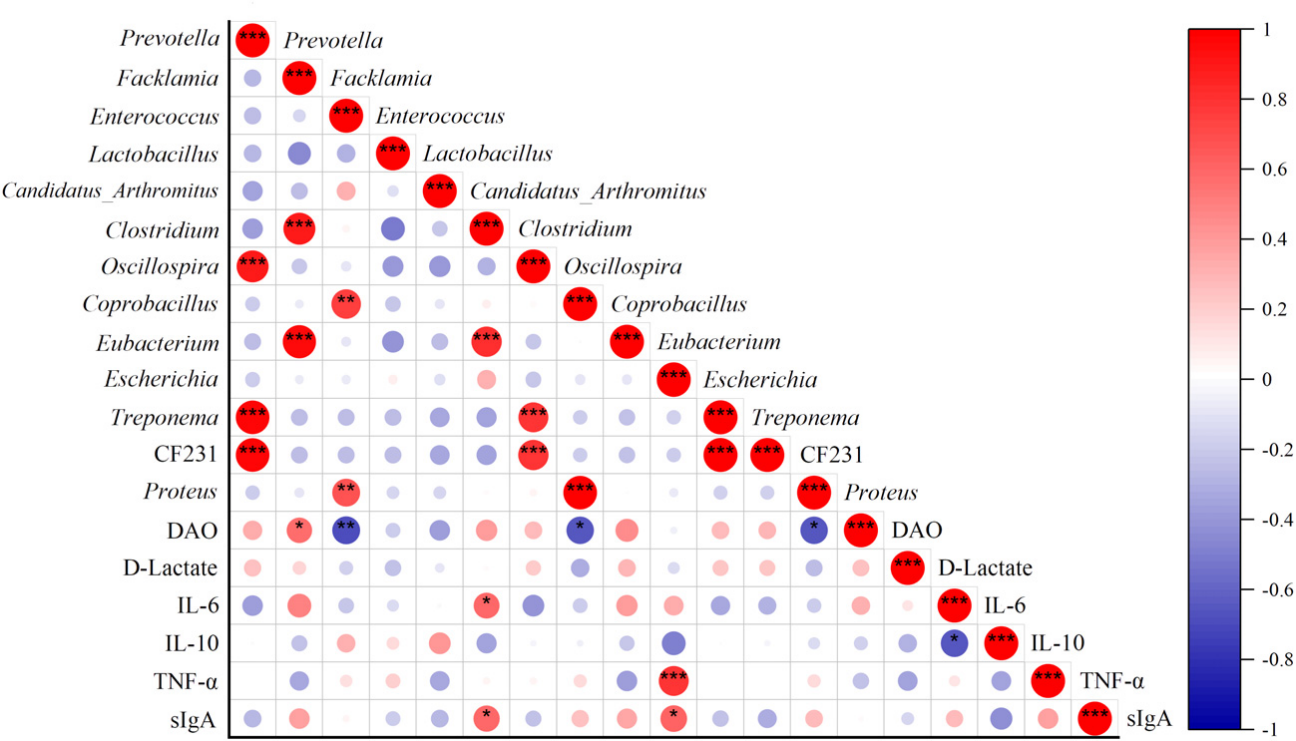
Correlations of specific genera with DAO, D-Lactate, sIgA, IL-6, IL-10, and TNF-α. Red points represent a positive correlation, and purple points represent a negative correlation. ^*^ *P* < 0.05, ^**^ *P* < 0.01, and ^***^ *P* < 0.001.

Correlation analysis was used to establish the association between MAM and biochemical indices. The heatmap demonstrated that *Clostridium* was strongly positively related to the levels of IL-6 (*P* = 0.029) and sIgA (*P* = 0.025), while *Escherichia* was significantly positively related to the levels of TNF-α (*P* = 0.00086) and sIgA (*P* = 0.020). Moreover, DAO was positively related to *Facklamia* (*P* = 0.035) and negatively related to *Enterococcus* (*P* = 0.0072), *Coprobacillus* (*P* = 0.011), and *Proteus* (*P* = 0.012) (**Figure 7**). The results indicated that the increase in abundance of *Clostridium* and *Escherichia* is associated with inflammation and immune response of intestinal mucosa.

## Discussion

Many studies in gut microbiota research have focused on the colon and feces. However, recent interest has begun to move toward the small intestine. Evidence suggested that disruptions to the small intestinal microbiota altered the host’s susceptibility to non-infectious enteric diseases (Shealy et al., 2024). Mucosal microorganisms adhere to the host mucosa closely and form biofilms that inhibit the proliferation of other bacteria (Galley et al., 2014). Therefore, the changes in diversity, structure, and function of MAM may alter the mucosal barrier. Studies have shown that the damage to intestinal mucosa resulted in the entry of bacteria and their derived toxins into vital tissues and organs through the bloodstream (Cox et al., 2017). Meanwhile, this may trigger the expansion of colon-like microorganisms in the small intestine (Burns et al., 2022). It has been reported that patients with inflammatory bowel disease exhibited intestinal microbiota dysbiosis characterized by an increased number of MAM and a decrease in the overall biodiversity (Palmela et al., 2018). In this study, an abnormal increase of small intestinal bacteria in the AAD mice was observed, suggesting that mixed antibiotic intervention-induced small-intestinal microbiota dysbiosis.

Generally, Proteobacteria is a minor phylum in a healthy gut. However, a bloom of Proteobacteria, especially an increased abundance of *γ*-Proteobacteria, is associated with many diseases. Family Enterobacteriaceae in Proteobacteria have a high affinity for mucin, which can destroy the intestinal barrier and trigger a systemic inflammatory response by degrading mucin (Raimondi et al., 2021). Increased Enterobacteriaceae in the small intestine was related to thinner mucus barriers (Bamba et al., 2023). Therefore, Proteobacteria is regarded as a microbial signature of intestinal mucosal epithelial dysfunction (Shin et al., 2015; Litvak et al., 2017). In this study, phylum Proteobacteria, family Enterobacteriaceae, and genus *Escherichia* were enriched in the small intestinal mucosa after mixed antibiotic treatment, indicating that mixed antibiotics induced intestinal barrier destruction in mice and increased the risk of pathogen invasion. These results were proved by the following detection of intestinal mucosal barrier indices. In detail, the levels of DAO, D-Lactate, and sIgA were increased after mixed antibiotic treatment. Both QWBZP decoction and QWBZP-TG regulated these indices but exhibited different strengths, which may be related to their different regulated effects on the MAM.

*Lactobacillus* is the most important probiotic bacteria of the gut microbiota. It combines with specific receptors on the surface of the intestinal mucosal epithelium to form a stable membrane structure and biological barrier, which can inhibit pathogenic bacteria by producing antibacterial substances or competing nutrients (Rastogi and Singh, 2022). Additionally, *Lactobacillus* is well known for suppressing inflammatory responses by inhibiting the expression of T-helper 17 (Th17) inflammatory cells and TNF-α (Ding et al., 2020). *Candidatus_Arthromitus*, commonly referred to as segmented filamentous bacteria (SFB), is one kind of commensal organism that attaches to the intestinal epithelium. Growing evidence supports that SFB promotes adaptive and innate immunity in mice through the differentiation and maturation of Th17 cells in the intestinal tract and the production of immunoglobulin A (IgA) (Shi et al., 2019; Hedblom et al., 2018). In this study, *Lactobacillus* and *Candidatus_Arthromitus* were two dominant bacteria in the intestinal mucosa of normal mice, and they maintained the integrity of the intestinal mucosal barrier through antagonistic action against other genera (**Figure 7**). The abundance of *Lactobacillus* and *Candidatus_Arthromitus* reduced sharply after mixed antibiotic treatment, which opened a door for other genera to flood the intestinal mucosa. QWBZP decoction was beneficial in promoting the proliferation of *Lactobacillus*, implying that QWBZP decoction remedied AAD by regulating the mucosa-associated core bacteria and commensal bacteria and then recovered the mucosal barrier. Of note, QWBZP-TG downregulated the abundance of *Lactobacillus* and *Candidatus_Arthromitus* in the AAD mice, suggesting that the influences of QWBZP-TG on *Lactobacillus* and *Candidatus_Arthromitus* were not the same as that of QWBZP decoction. Our previous studies showed that the polysaccharide, another dominant component in QWBZP decoction, did not recover the abundance of *Lactobacillus* and *Candidatus_Arthromitus* that was reduced by mixed antibiotics (Li et al., 2022). These findings showed the superiority of the combination of the TCM formula. Surprisingly, we found that the QWBZP decoction and QWBZP-TG promoted the proliferation of lumen-associated *Lactobacillus* in the AAD mice, and the intensity of this effect was more significant in the case of QWBZP-TG (Xie et al., 2022a). It suggested that the regulation effects of QWBZP-TG on the gut microbiota are niche-dependent.

*Prevotella* was a signature bacteria responding to QWBZP-TG treatment. Intriguingly, some of the reported findings were controversial regarding the beneficial or harmful properties of *Prevotella*. Some literature reported that *Prevotella* was dominant in the intestines of people who eat a healthy diet rich in vegetables and fruits. *Prevotella* helps to decompose fiber and produce health-promoting compounds such as short-chain fatty acids (Fehlner-Peach et al., 2019; De Filippis et al., 2019). Conversely, other researchers suggested that the enrichment of *Prevotella* was associated with Th17-related inflammatory diseases, hypertension, insulin resistance, and glucose intolerance (Huang et al., 2020; Larsen, 2017; Alpizar-Rodriguez et al., 2019; Li et al., 2017; Pedersen et al., 2016). Tett et al. (2019) found that *Prevotella* exhibited high genome diversity. Thus, it should be cautioned to discuss the role of *Prevotella* in the efficacy evaluation. It is well known that glycosides are composed of aglycones and sugar moieties, and the two groups are connected by glycosidic bonds. We suggested that *Prevotella* enrichment in the intestinal mucosa after QWBZP-TG treatment helped to cleave the glycoside bone and metabolize QWBZP-TG, then the metabolites may benefit to improve inflammation of AAD mice. However, further studies are needed to prove this deduction.

In TCM, the main type of AAD is kidney-yang deficiency syndrome (Chen et al., 2024). Recently, the characteristic of gut microbiota in diarrheal mouse modeling with kidney-yang deficiency syndrome induced by trimethylamine-N-oxide (TMAO) was evaluated by Xie et al. (2024). This research highlighted that diarrheal mice with kidney-yang deficiency syndrome exhibited increased richness and diversity in the small intestinal microbiota compared with the normal mice (Xie et al., 2024). In addition, TMAO could activate inflammatory responses and cytokines, which contribute to diarrhea with kidney-yang deficiency syndrome through the “gut-kidney axis” (Xie et al., 2024; Zhou et al., 2024a, Zhou et al., 2024b). The gene for trimethylamine production, such as carnitine oxygenase (CntA), was present in *Escherichia* (Rath et al., 2020). Furthermore, the level of TMAO was significantly positively correlated with IL-6 (Guo et al., 2024). The upregulated expression of the TNF-α and IL-6 increased the permeability of vascular endothelial cells, leading to intestinal fluid extravasation, which can cause diarrhea (Chen et al., 2022). In this study, the changes of MAM and cytokines in the small intestine of AAD mice were similar to those of diarrheal mice with kidney-yang deficiency syndrome. However, whether antibiotics were involved in diarrhea by mediating the “gut-kidney axis” needs further research.

## Conclusion

**Figure 8:**
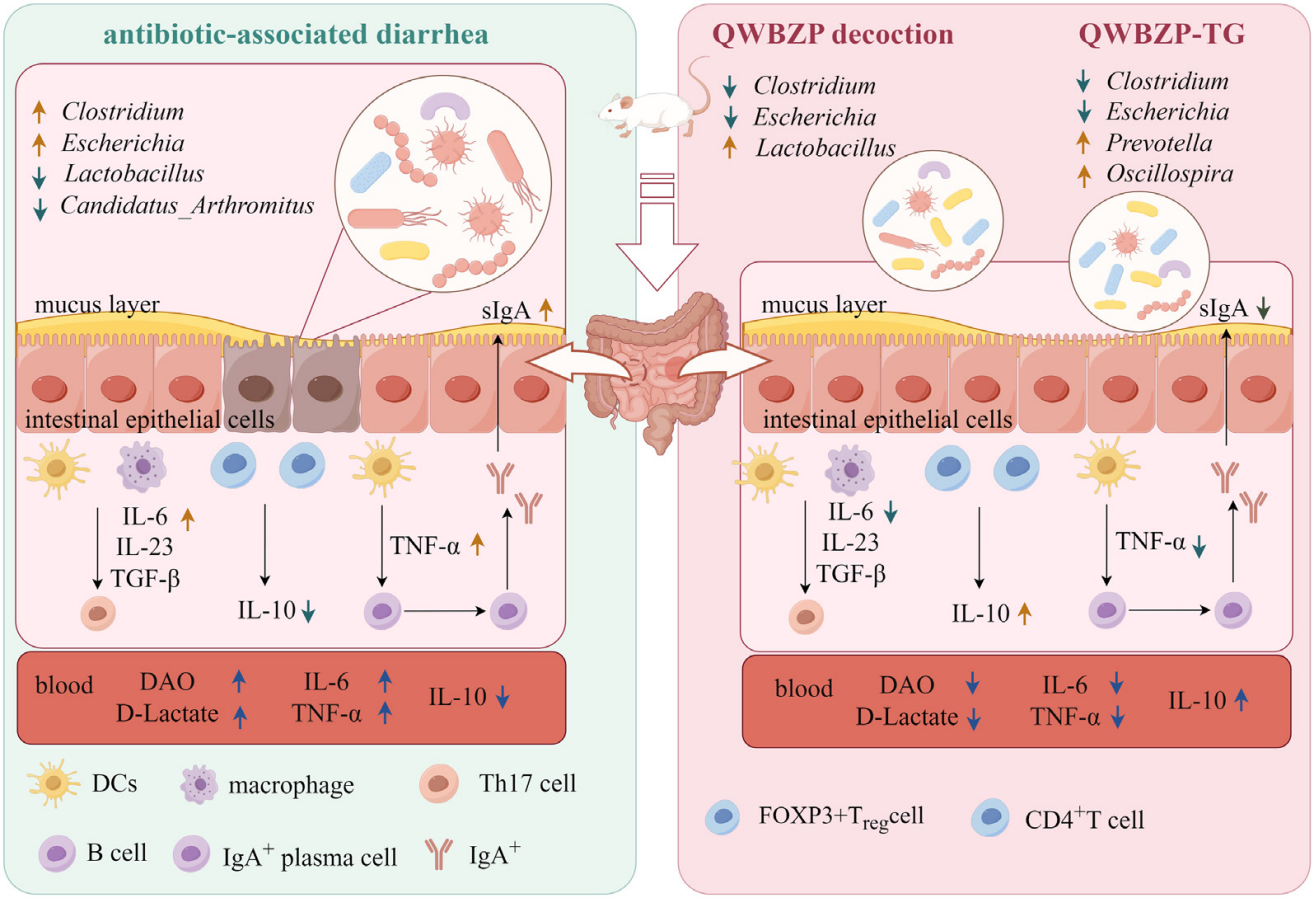
The possible mechanisms of QWBZP decoction and QWBZP-TG in the treatment of antibiotic-associated diarrhea. Figdraw (https://www.figdraw.com/#/) was used to create the figure.

QWBZP-TG significantly changed the diversity, structure, and abundance of MAM in the small intestine of AAD mice. The effect of QWBZP-TG on mucosa-associated *Lactobacillus* was opposite to that of QWBZP decoction, while the suppressed effects of QWBZP-TG on diarrhea-associated *Escherichia* and *Clostridium* were stronger than that of QWBZP decoction. *Prevotella* was the signature bacteria that responded to QWBZP-TG. Both QWBZP decoction and QWBZP-TG modulated inflammatory factors and immunological barriers (**Figure 8**). This study provided a new idea on the efficacy research of TCM formulas and their bioactive compounds.

## Data Availability

The data presented in the study are deposited in the NCBI repository, accession number PRJNA975495.
