# Supplementary material for: Co-frequency or contrary? The effects of Qiwei Baizhu Powder and its bioactive compounds on mucosa-associated microbiota of mice with antibiotic-associated diarrhea
Source: Front Cell Infect Microbiol. 2024 Oct 28;14:1483048. doi: 10.3389/fcimb.2024.1483048 (PMC11551125; doi:10.3389/fcimb.2024.1483048)
Supplement: Supplementary file 1 [file Table1.docx]

**Supplementary Table 1**

Identification of chemical constituents of QWBZP-TG

| No. | Identification | Formula | Relative amount (%) |
| --- | --- | --- | --- |
| 1 | puerarin | C_21_H_20_O_9_ | 7.25 |
| 2 | 3'-methoxypuerarin | C_22_H_22_O_10_ | 5.48 |
| 3 | 3'-hydroxypuerarin | C_21_H_20_O_10_ | 3.79 |
| 4 | 6''-*O*-xylosylpuerarin | C_26_H_28_O_13_ | 3.62 |
| 5 | daidzin | C_21_H_20_O_9_ | 2.31 |
| 6 | isoacteoside | C_29_H_35_O_15_ | 2.19 |
| 7 | acteoside | C_29_H_35_O_15_ | 1.29 |
| 8 | genistein-8-*C*-apiosyl(1-6)-glucoside | C_26_H_28_O_14_ | 0.92 |
| 9 | formonononetin-8-*C*-glucoside-*O*-xyloside | C_27_H_30_O_13_ | 0.76 |
| 10 | daidzein | C_15_H_10_O_4_ | 0.66 |
| 11 | ginsenoside Rg_1_ | C_42_H_72_O_14_ | 0.49 |
| 12 | glycyrrhizic acid | C_42_H_62_O_16_ | 0.43 |
| 13 | Isoliquiritin apioside or isomer | C_26_H_30_O_13_ | 0.41 |
| 14 | genistein-8-*C*-glucoside | C_21_H_20_O_10_ | 0.39 |
| 15 | pueroside A | C_29_H_34_O_14_ | 0.38 |
| 16 | ginsenoside Rk_3_ | C_36_H_60_O_8_ | 0.25 |
| 17 | 4'-*O*-methoxypuerarin | C_22_H_22_O_9_ | 0.21 |
| 18 | ginsenoside Ro | C_48_H_76_O_19_ | 0.18 |
| 19 | ginsenoside 20(*R*)-Rg_3_ | C_42_H_72_O_13_ | 0.17 |
| 20 | ginsenoside F_1_ | C_36_H_62_O_9_ | 0.16 |
| 21 | genistin | C_21_H_19_O_10_ | 0.15 |
| 22 | 3'-methoxypuerarin-6''-*O*-*β*-Apionoside | C_27_H_30_O_14_ | 0.12 |
| 23 | liquiritin^*^ | C_21_H_22_O_9_ | 0.12 |
| 24 | puerarin-7-*O*-glucoside | C_27_H_30_O_14_ | 0.11 |
| 25 | daidzein-4',7-*O*-glucoside | C_29_H_32_O_18_ | 0.11 |
| 26 | 3'-hydroxypuerarin xyloside | C_26_H_28_O_14_ | 0.07 |
| 27 | formonononetin | C_16_H_12_O_4_ | 0.07 |
| 28 | Liquiritin apioside^*^ | C_26_H_30_O_13_ | 0.06 |
| 29 | genistein | C_15_H_10_O_5_ | 0.06 |
| 30 | apigenin | C_15_H_10_O_5_ | 0.06 |
| 31 | ginsenoside Rc | C_53_H_90_O_22_ | 0.04 |
| 32 | 4',6-dimethoxyisoflavone-7-*O*-glucoside | C_23_H_24_O_10_ | 0.03 |
| 33 | isoliquiritin | C_21_H_22_O_9_ | 0.03 |
| 34 | licorice saponin G_2_ | C_42_H_62_O_17_ | 0.02 |
| 35 | leucosceptoside A | C_30_H_37_O_15_ | 0.02 |
| 36 | naringenin | C_15_H_12_O_5_ | 0.02 |
| 37 | licorice saponin A_3_ | C_48_H_72_O_21_ | 0.01 |
| 38 | ginsenoside Re_1/2/3_ | C_48_H_82_O_19_ | — |
| 39 | ginsenoside Ⅰ/Ⅱ | C_48_H_82_O_20_ | — |
| 40 | ginsenoside Re | C_48_H_82_O_18_ | — |
| 41 | ginsenoside Rh_1_ | C_36_H_62_O_9_ | — |
| 42 | ginsenoside Rf | C_42_H_72_O_14_ | — |
| 43 | ginsenoside 20(*S*)-Rg_2_ | C_42_H_72_O_13_ | — |
| 44 | ginsenoside 20(*R*)-Rg_2_ | C_42_H_72_O_13_ | — |
| 45 | ginsenoside Rb_2_/Rb_3_ | C_53_H_90_O_22_ | — |
| 46 | ginsenoside Rd | C_48_H_82_O_18_ | — |
| 47 | ginsenoside Rg_9_ | C_42_H_70_O_13_ | — |
| 48 | ginsenoside F_2_ | C_42_H_72_O_13_ | — |
| 49 | ginsenoside Rg_6_ | C_42_H_70_O_12_ | — |
| 50 | ginsenoside 20(*S*)-Rg_3_ | C_42_H_72_O_13_ | — |
| 51 | ginsenoside F_4_ | C_42_H_70_O_12_ | — |
| 52 | ginsenoside Rs_4_ | C_44_H_72_O_13_ | — |
| 53 | soyasaponin Ba | C_48_H_78_O_19_ | — |
| 54 | azukisaponin Ⅱ | C_42_H_68_O_14_ | — |
| 55 | 22*β*-acetoxylglycyrrhizic acid | C_44_H_64_O_18_ | — |
| 56 | 3'-hydroxypuerarin-4'-*O*-glucoside | C_27_H_30_O_15_ | — |
| 57 | puerarin-4'-*O*-*β*-*D*-glucoside | C_27_H_30_O_14_ | — |
| 58 | 3'-methyoxy-4'-*O*-glucosyl-puerarin | C_28_H_32_O_15_ | — |
| 59 | mirificin | C_26_H_28_O_13_ | — |
| 60 | 3'-methoxydaidzin | C_22_H_22_O_10_ | — |
| 61 | 5'-hydroxyl oninin | C_22_H_22_O_10_ | — |
| 62 | 6''-*O*-acetyl daidzin | C_23_H_22_O_10_ | — |
| 63 | ononin | C_22_H_22_O_9_ | — |
| 64 | apigenin-7-*O*-*β*-*D*-glucopyranoside | C_21_H_20_O_10_ | — |
| 65 | glycitein | C_16_H_12_O_5_ | — |
| 66 | 3'-methoxydaidzein | C_16_H_12_O_5_ | — |
| 67 | irisolidone | C_17_H_14_O_6_ | — |
| 68 | calycosin | C_16_H_12_O_5_ | — |

Note: '—' means the relative amount < 0.01%.
